# Religion and ethnicity interaction as a predictor of male fertility in Nigeria: Evidence from a national representative sample

**DOI:** 10.1371/journal.pone.0296983

**Published:** 2024-01-25

**Authors:** Ayo Adebowale, Martin Palamuleni

**Affiliations:** 1 Department of Epidemiology and Medical Statistics, Faculty of Public Health, University of Ibadan, Ibadan, Nigeria; 2 Population and Health Research Entity, Faculty of Humanities, North-West University, Mafikeng, South Africa; Public Library of Science, UNITED KINGDOM

## Abstract

High fertility constitutes a challenge to men’s health conditions in Nigeria, a low-income country. Religion and ethnicity are central to the current Male Fertility (MF) level in Nigeria. This study determined the relationship between Male Fertility (MF) and Religion Ethnic Interaction (REI) amidst other socio-demographic characteristics in Nigeria. Nigeria Demographic and Health Survey data, 2018 was used. Men aged 15–59 years [n = 8,786] were sampled using a multi-stage approach. Fertility was measured by the number of Children Ever Born (CEB). REI was generated using the combination of religion and ethnic groups; Hausa/Fulani Muslim, Igbo Christian, Yoruba Christian, and Yoruba Muslim. However, Hausa/Fulani Christians and Igbo Muslims were excluded from this study because a few men in these categories were available in the original sample. Weighted data were analyzed using the Negative Binomial (NB) model (α_0.05_). The mean age and CEB of the men were 32.9±12.0 years and 3.18±4.4 respectively. The mean CEB among men aged 45–59 years was highest among Hausa/Fulani Muslims (x = 11.57±5.98), but least among Yoruba Christians (x = 4.44±2.67). About 33.4% of the Hausa/Fulani Muslims had had ≥5 children, while 13.7% were reported among the Yoruba Christian men (p<0.001). The fertility Incidence Rate Ratio (IRR) was higher among Hausa/Fulani Muslims, but less among Igbo Christians, and Yoruba Christians than Yoruba Muslims. Restricting the analyzed data to only monogamous men revealed no significant differences in the fertility IRR of Yoruba Christians and Yoruba Muslims, but the fertility IRR was significantly higher among the Hausa/Fulani Muslims than Yoruba Muslims. A disparity exists in MF across the REI groups with the Hausa/Fulani Muslims being the major contributors to high MF. Therefore, bridging the gap in access to fertility control measures and programmes that might have resulted from religion and ethnic differences will reduce male fertility level in Nigeria.

## Introduction

Nigeria launched its first population policy in 1988 and revised it in 2022 with an emphasis to reduce the number of children to an average of 4 per family and address persistent high fertility rate in Nigeria with the view to improving population health [1, 2]. Nigeria has the largest population in Africa, seventh globally and the population is still growing. It has been projected that by the year 2050, Nigeria will be the third most populous country worldwide if the current trend in population growth rate is sustained over the period [3]. With a population figure of over 215 million in 2022, and a growth rate of 2.5, the population is expected to double in 28 years [4]. Unfortunately, The total fertility rate (5.3) is high, the Contraceptive Prevalence Rate (CPR) among married women is low (17.0%), and the unmet need for contraceptives (18.9%) remains high in the country [5]. The high fertility level in Nigeria narrows prospects of achieving the 2030 Sustainable Development Goals (SDGs), and slows its demographic transition [6]. The sustenance of high fertility calls for research aimed at determining the segment of Nigerian society that should be prioritized for fertility reduction interventional practices.

The present Nigerian society, like most of Africa, is patrilineal where men are involved in virtually all household decisions including the number of children a woman should bear and the timing of such births [7, 8]. The inheritance or rule descends through the family’s males. The tradition underscores the provision of household needs like food, shelter, health, and daily expenses as the obligation of men [8]. Such responsibilities could be overwhelming in the face of poor income, poverty, and harsh economic condition in Nigeria. Accordingly, it is not unlikely that the number of children fathered by a man shapes his career goals and well-being just like that of women. In most fertility studies worldwide, numerous family questions, fertility risks, and consequences have mainly been documented concerning women [9–13]. The patterns, levels, dynamics, and determinants of male fertility have remained understudied research mainly due to data constraints and methodological challenges [14]. Studying male fertility is important going by the roles of men in reproductive activities and society. Knowledge of the prevalence and determinants of male fertility can complement the wide available evidence of female fertility [14]. Religion, ethnicity, and men’s roles are variables central to fertility dynamics. Despite the importance of these variables on fertility, most existing research on the relationship between religion, ethnicity, and fertility in Nigeria focused on women [10–12].

Researchers have documented consistent fertility differences between Christians and Muslims. In these studies, higher fertility among Muslims was attributed to lower CPR, polygamy which promotes birth competition among wives, and also education, income, political, and social differences [10–12, 15]. However, ethnic identity plays an important role in the relationship between religion and fertility. Theoretically, the basis for studying the association between religion and fertility is a reflection of the focus on each religious organizational difference. This is because religion; spreads norms about specific fertility-related behaviors, has ways of enforcing conformity to these norms among its members, and is influential when its members feel a strong sense of religious cohesion [16]. Specific religious teachings about fertility-related behavior and active enforcement of these norms are pertinent components of the connection between religion and fertility [16]. Religious identities in Nigeria may be seen as cultural and religious doctrine most at times include attitudes and values about family issues including fertility [17].

While ethnicity is used to classify people into social groups according to their common culture, language, and heritage, religion refers to the specific beliefs of people. Ethnic groups have traditions and other life-styles attributes they adhere to help preserve the shared heritage and fit the context in which they live [18]. Consequently, individuals who belong to the same religious denomination, but either from the same (intra-) or different (inter-) ethnic group(s) may exhibit different fertility risks and childbearing behaviors [17]. Arguably, we perceive that analyses based on the national composition of individuals in the same religious group without the reflection of their ethnic identity may either distort the outcome or lead to a spurious interpretation of the findings.

The main independent variable in this study is Religion Ethnicity Interaction (REI) and this was created using a mixture of the religious and ethnic categories. However, the apparatus connecting REI and male fertility has not been investigated using Nigeria data as established in the current study. We proceeded under the hypothesis that there is no significant link between REI and male fertility. The objectives of this study are to determine the prevalence of high fertility (≥5 CEB) among Nigerian men by REI and examine the relationship between male fertility and REI amidst other socio-demographic characteristics. The findings will guide the policymakers and fertility planners in their decision to target REI as an important variable for fertility reduction strategies in Nigeria.

## Materials and methods

### Study area

The study was conducted in Nigeria, the country with the largest population in Africa. According to the world population data sheet, the population of Nigeria was 218.5 million with a growth rate of 2.4%, and a life expectancy of 54 years in 2022 [4]. The men aged 15–59 years constitute 26.5% of the Nigerian population [18]. There are 36 states including Federal Capital Territory (FCT), Abuja in Nigeria, and geographically delineated into six geopolitical zones. While males’ school enrolment is still more than females’ in proportion, a significant proportion of men have no formal education. In Nigeria, the most basic form of identity is ethnicity and religion is another important form of identity [19]. While Muslims and Christians can be found in all parts of Nigeria, the strongest footholds of the Muslims are among the Hausa/Fulani and the Yoruba. Christianity is most prevalent in the southern part of Nigeria, the vast majority of Igbo are Christians. Neither Christianity nor Islam is free of the influence of traditional religious practices.

### Study design and population

This cross-sectional design study utilized the 2018 Nigeria Demographic and Health Survey (NDHS) data [5]. The sample was nationally representative and a multi-stage sampling approach was used for the selection of 13,311 men aged 15–59 years [5].

### Sampling strategy

The sampling frame used was based on the updated 2006 Nigeria Population and Housing Census frame. Administratively, Nigeria is divided into states. Each state is subdivided into local government areas (LGAs), and each LGA is divided into localities and each locality is subdivided into enumeration areas (EAs) which was the primary sampling unit (PSU). A two-stage sampling technique including cluster and systematic sampling was used to randomly select 42,000 households across Nigeria. Sampling weights were generated separately for each sampling stage and each cluster. The men’s survey was conducted in one-third of the sample households, and all men aged 15–59 years in these households were included. The Men’s Questionnaire used for the 2018 NDHS was adapted to reflect the population and health issues relevant to Nigeria [5]. For further exploration of the sampling procedure of this study, explicit information is available at www.measure.dhs.

### Variable definition

**Fig 1:**
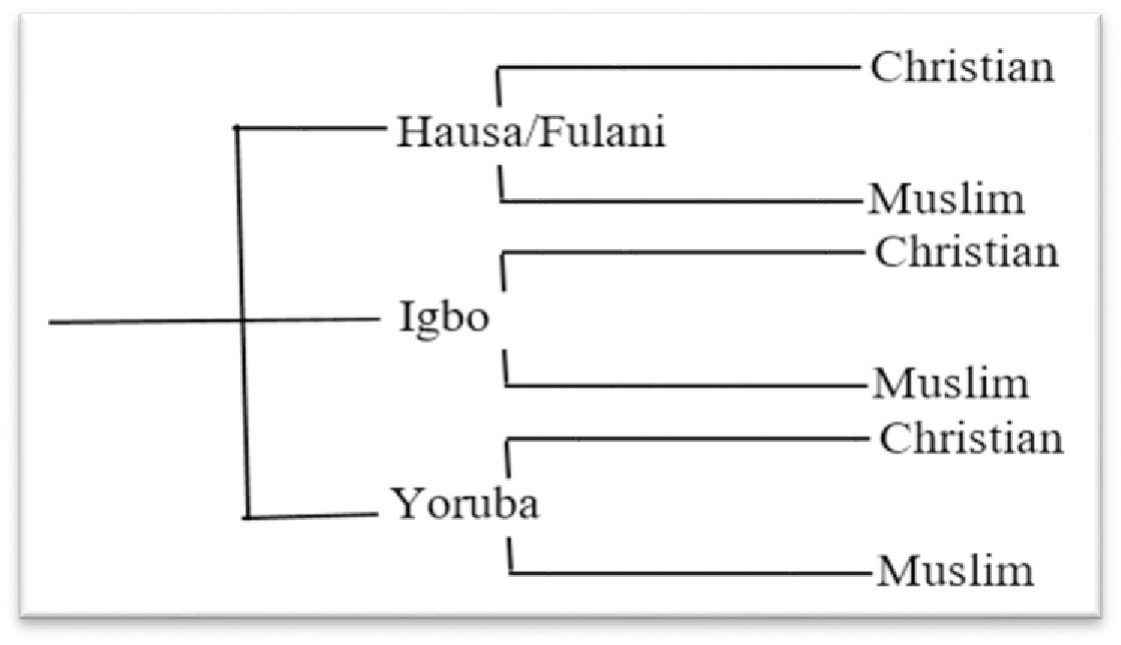
Tree diagram showing the generation of REI.

The dependent variable was fertility measured by the number of children ever born (CEB) as reported by men irrespective of the number of women that a man fathered a child with. All men aged 15–59 years in the sample were analyzed. This variable was used majorly in quantitative terms but also used as a categorical variable: 0, 1–2, 3–4, 5+. In the context of this study, men who had had at least 5 children are regarded as having high fertility. The main independent variable was REI created as follows. There are three main religious groups in Nigeria; Christianity, Islam, and Traditional, but Christianity and Islam are mostly common with Traditional religious members constituting a very small proportion. The sample for this study reflected this distribution, therefore, the men who belong to traditional religious denominations were excluded from the analysis. Thus, only men who are either Christian or Muslim were included in the study. Nigeria also has several Ethnic groups, but the major ethnic groups in the country are Hausa/Fulani, Igbo, and Yoruba with other ethnic groups excluded. Therefore, REI was generated using the combination of religion and ethnic groups (Fig 1).

Thus, the sample space, S = {Hausa/Fulani Christian; Hausa/Fulani Muslim; Igbo Christian; Igbo Muslim; Yoruba Christian; Yoruba Muslim}. In the original sample, very few men reported that they were Hausa/Fulani Christian and Igbo Muslim. This constituent shows the true reflection of ethnic religion composition in Nigeria, the Hausa/Fulani are predominantly Muslim, while the Igbos are largely Christian. Therefore, these two groups of men were excluded from the analyzed sample. The main independent variable, REI has four categories: Hausa/Fulani Muslim; Igbo Christian; Yoruba Christian; Yoruba Muslim. Thus, the exclusion of individuals based on the criteria specified above led to a reduction in the sample size from 13,311 to 8,786 with Hausa/Fulani Muslim, Igbo Christian, Yoruba Christian, and Yoruba Muslim constituting 53.2%(4660), 22.5%(1977), 12.2%(1069), and 12.3%(1080) of this sample respectively. A systematic error might have been created due to the exclusion of 4255 men from the analysis, but the anticipated bias will be minimized because of the robustness and representativeness of the data. Other independent variables used as a control to ascertain the relationship between REI and Fertility are Age, Place of residence, level of education, Wealth index, Age at first birth, Marital status, Age at first cohabitation, Number of marital unions, and Family type.

### Data analysis

**Fig 2:**
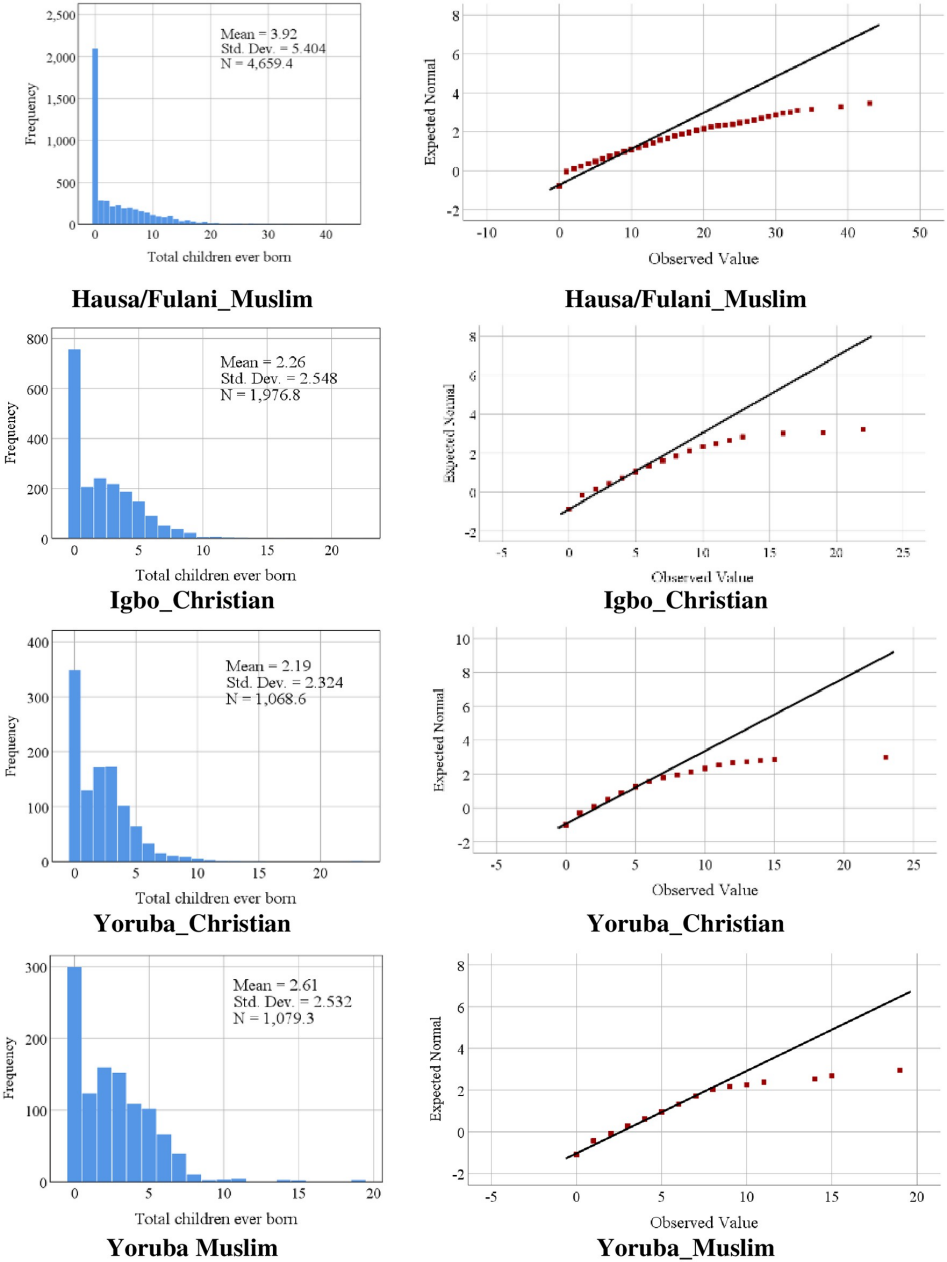
Histogram and Q–Q normality plot of children ever born by Religion and Ethnic Interaction (REI).

The weighted data were analyzed using SPSS software version 25.0. Frequency, percentages, and mean were used to describe the data. Histogram and Q-Q plot was used to examine zero composition and normality of fertility across the REI groups (Fig 2).

Fertility was presented by line graphs and dot plots. A chi-square test was performed to examine the association between fertility and REI. At the multivariate level of analysis, the Negative Binomial Mixed Model (NBM), a class of the Generalized Linear Model (GLM) was used to determine the relationship between fertility and REI. One useful approach to circumvent the problem of over-dispersion in the fertility count Y (μ = 3.18, σ = 4.36, σ2 = 19.03) in the current study is the use of NBM in which the variance is a function of mean and dispersion parameter γ with varY = μ+μ2γ [20]. All robustness checks indicate that REI is relevant to the explanation of male fertility in Nigeria and that the NBM regression is the model of best fit for establishing the association between REI and male fertility in Nigeria. We used the model to test for connections between confounding and predictor variables on fertility based on the assumptions of linearity, homoscedasticity, and normality.

The NBM is a mixture of both the gamma and Poisson model with probability density function;

$$f\left(y_{i}/x_{i}\right)=\frac{\Gamma \left(y_{i}+\gamma \right)}{y_{i}!\Gamma (\gamma )}{\left(\frac{\gamma }{\gamma +\mu_{i}}\right)}^{\gamma }{\left(\frac{\mu_{i}}{\gamma +\mu_{i}}\right)}^{y_{i}},\;\gamma >0$$

NBM has been interpreted with the use of odds ratios with a 95% Confidence interval. The selection of NBM among other classes of GLM was based on the diagnostic check performed on the outcome variable.

Four models were used, and an unadjusted model involved the inclusion of only the dependent and each of the independent variables in the equation at a time. The second and third models included only non-marital (Age, Place of residence, level of education, Wealth index) and marital (Age at first birth, Marital status, Age at first cohabitation, Number of marital unions, and Family type) related variables respectively, while the fourth model was the full model. The level of significance was set at 5.0%. However, multicollinearity was examined between the related variables in the model.

### Ethical consideration

I confirm that the study was implemented under relevant guidelines and regulations. Secondary data was used for this study and as such, permission to use the data was sought and granted by the data originator. However, NPC and ICF Macro obtained ethical approval to conduct the survey from the National Ethical Review Committee (NREC), and at the point of data collection, informed consent was also obtained from the respondents before the conduct of the interview. The respondents were assured of the anonymity of the information they provided. The possible identifier that could be used to track each respondent to the information they provided was removed from the data before use.

## Results

**Fig 3:**
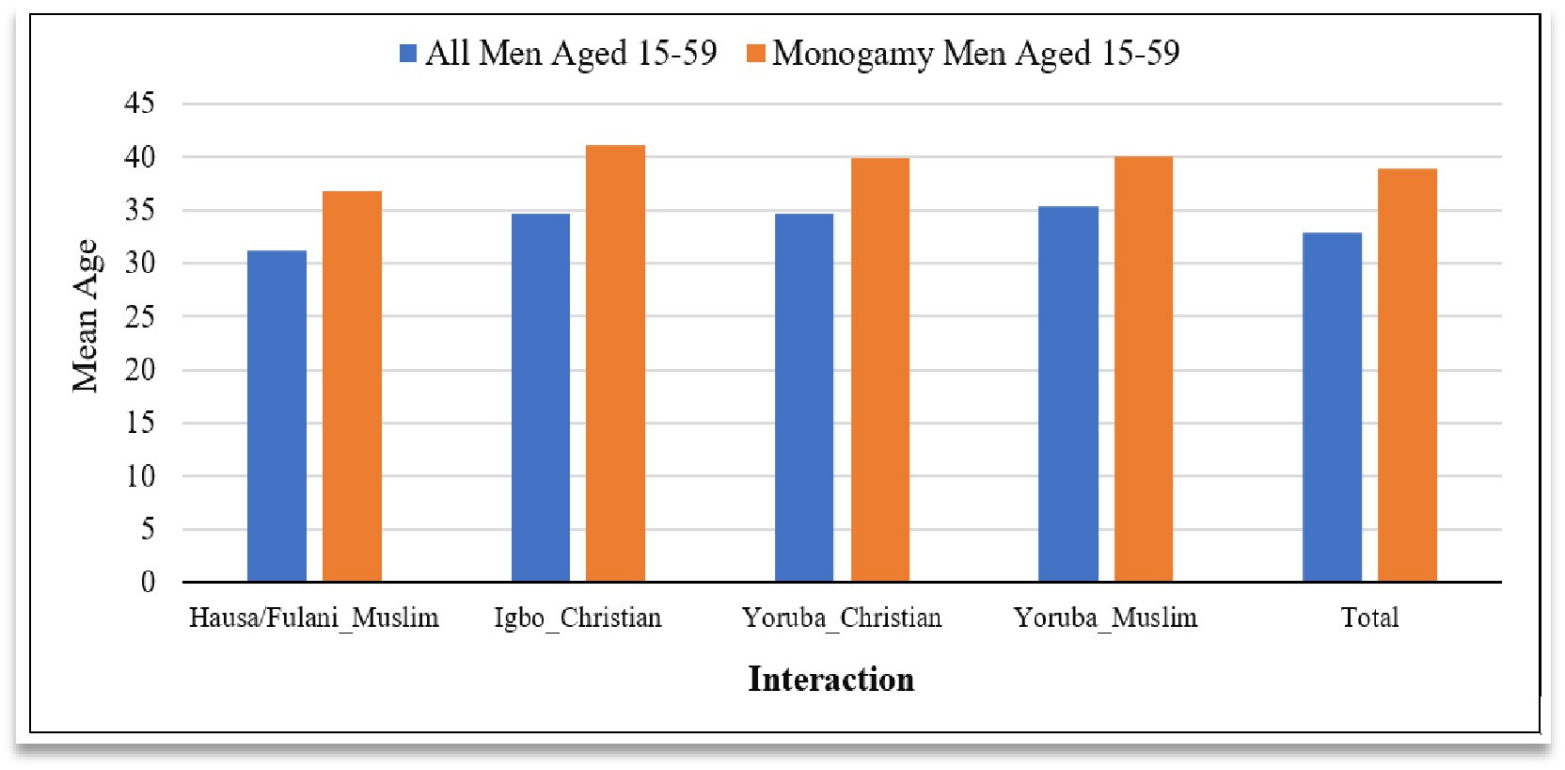
Mean age of men in years according to REI.

Among all the men studied, their mean age was 32.9±12.0 years. Across the REI groups, the mean age was 31.1±12.1 years among Hausa/Fulani Muslim, 34.7±11.9 years in Igbo Christian, 34.7±11.4 years in Yoruba Christian, and 35.3±12.0 years in Yoruba Muslim. The mean age was consistently higher among the monogamist men than the combined monogamist and polygamist men. Among the monogamist men, the mean age in years was highest among the Igbo Christians (41.13±8.29), followed by Yoruba Muslims (40.02±7.92), then, Yoruba Christians (39.98±8.19), and least among Hausa/Fulani Muslims (36.81±9.51) (Fig 3).

The distribution of the study subjects according to children ever born (CEB) by socio-demographic characteristics is presented in Table 1. About 33.4% of the Hausa/Fulani men who are Muslim had had at least five children, while 18.7%, 13.7%, and 21.8% were reported among the Igbo Christian, Yoruba Christian, and Yoruba Muslim men respectively (p<0.001). As expected, the percentage of men who have at least five children increases consistently as the age group increases. It increases significantly from 0.1% among men in the age group 15–24 years to 68.1% among men in the age group 45–59 years. A higher percentage of men who have given birth to five children and above was found in the rural area (30.2%) compared to the urban area (19.5%) p<0.001. The percentage of men who have given birth to at least five children was highest among men with no formal education (42.1%) and this reduces steadily to 15.8% for those who have tertiary education. This pattern was observed in variables such as wealth index, age at first birth, and age at first cohabitation. While a strikingly higher percentage of men with CEB≥5 was reported by those in the polygamous family (85.8%) than those who have only one wife (32.8%) p<0.001.

**Table 1:** Percentage distribution of men according to Children Ever Born (CEB) by background characteristics.

Background Characteristics	All MEN Aged 15–59 years			Monogamist MEN Aged 15–59 years		
	CEB	Total	$\chi_{value}^{2}$ *(p value)*	CEB	Total	$\chi_{value}^{2}$ *(p value)*
	5+			5+		
**Total**	26.3(2307)	8783		33.0(1511)	4583	
**Ethnic Religion Interaction**			**786.1** *			**346.3** *
Hausa/Fulani Muslim	33.4 (1558)	4660	*(* ***<0*.*001*** *)*	44.3(872)	1969	*(* ***<0*.*001*** *)*
Igbo Christian	18.7 (370)	1977		28.6(352)	1229	
Yoruba Christian	13.5 (144)	1069		16.8(117)	698	
Yoruba Muslim	21.8 (235)	1080		24.7(170)	687	
**Age**			**7041.3** *			**1341.7** *
15–24	0.1(2)	2560	*(* ***<0*.*001*** *)*	1.3(2)	155	*(* ***<0*.*001*** *)*
25–34	7.9(170)	2157		8.8(114)	1296	
35–44	39.9(906)	2273		32.9(600)	1822	
45–59	68.5(1229)	1793		60.7(794)	1309	
**Place of Residence**			**259.2** *			**132.2** *
Urban	20.1(939)	4672	*(* ***<0*.*001*** *)*	27.1(720)	2652	*(* ***<0*.*001*** *)*
Rural	33.2(1367)	4112		40.9(790)	1930	
**Level of Education**			**933.5** *			**376.3** *
None	42.6(952)	2237	*(* ***<0*.*001*** *)*	47.8(529)	1106	*(* ***<0*.*001*** *)*
Primary	40.9(540)	1320		45.3(373)	824	
Secondary	15.7(597)	3804		25.7(461)	1795	
Higher	15.2(217)	1423		17.3(148)	857	
**Wealth Index**			**520.6** *			**280.3** *
Poor	35.8(1004)	2802	*(* ***<0*.*001*** *)*	46.7(580)	1241	*(* ***<0*.*001*** *)*
Middle	29.3(734)	2505		37.9(470)	1241	
Rich	16.4(569)	3479		21.9(460)	2100	
**Age at First Birth**			**296.7** *			**159.2** *
<25	57.7(989)	1713	*(* ***<0*.*001*** *)*	47.0(572)	1218	*(* ***<0*.*001*** *)*
25–34	40.6(1170)	2883		33.5(812)	2424	
35–59	21.3(147)	690		19.2(126)	657	
**Marital Status**			**7497.8** *			N.A
Never Married	0.0(1)	3214	*(* ***<0*.*001*** *)*	N.A	N.A	(N.A)
Currently Married	41.8(2287)	5477		33.0(1510)	4581	
Previously Married	19.6(18)	92		N.E	N.E	
**Age at First Cohabitation**			**195.8** *			**123.5** *
<25	51.0(1158)	2271	*(* ***<0*.*001*** *)*	41.2(703)	1706	*(* ***<0*.*001*** *)*
25–34	36.8(999)	2714		29.7(697)	2345	
35–59	25.3(148)	586		20.9(111)	531	
**Number of Marital Unions**			**334.15** *			**328.0** *
Once	28.0(1147)	4095	*(* ***<0*.*001*** *)*	28.3(1135)	4017	*(* ***<0*.*001*** *)*
More than once	65.9(381)	578		66.4(375)	565	
**Family Type**			**1214.9** *			N.A
Monogamy	33.0(1510)	4581	*(* ***<0*.*001*** *)*	N.A	N.A	(N.A)
Polygamy	86.7(776)	895		N.A	N.A	

*Significant at 0.1%; N.A: Not Applicable; N.E: No Entry

**Fig 4:**
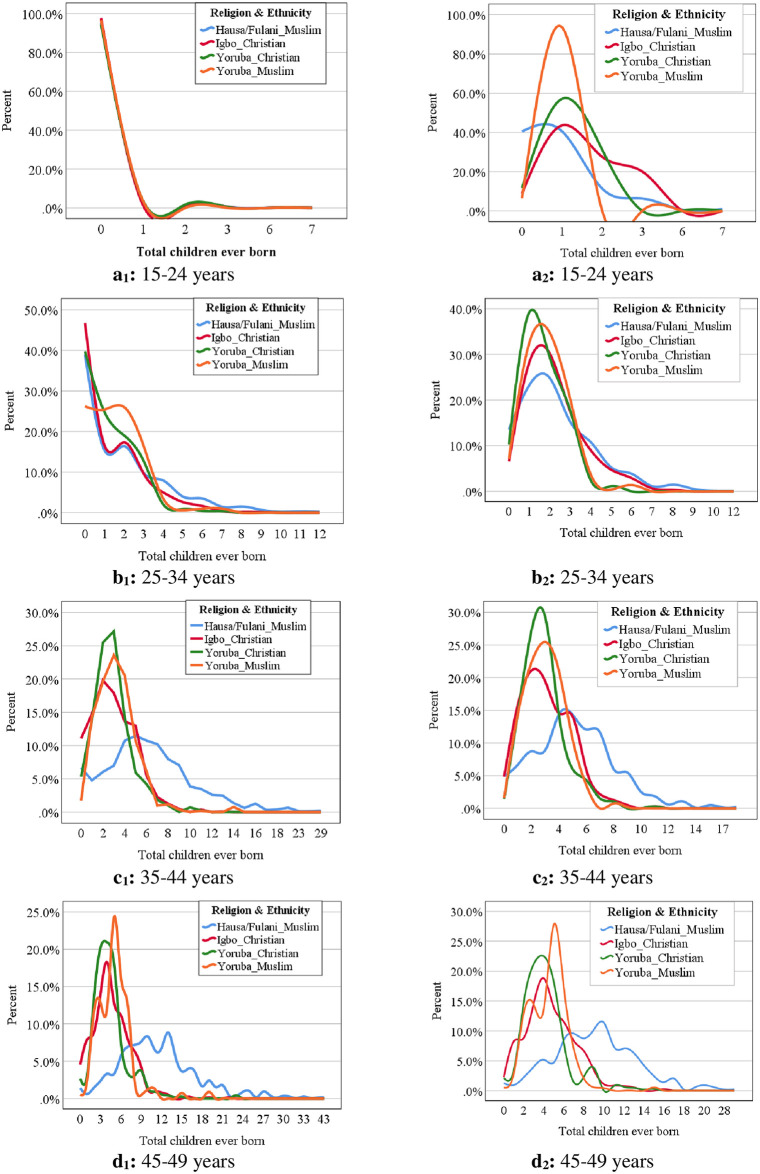
Percentage Distribution of Children Ever Born by Age in years according to Religion and Ethnic (REI) Interaction (All Men (4a_1_-4d_1_) and Monogamist Men (4a_2_-4d_2_)).

The graphs of the percentage distribution of CEB standardized by age in years according to REI are presented in Fig 4a_1_–4d_1_. For men in the age group 15–24 years, the pattern of the number of CEB was similar across the REI groups (Fig 4a_1_). However, in the higher age groups, the data show that Hausa/Fulani men who are Muslim have a higher proportion of their members having more children than any other REI groups and the difference was more pronounced among older men (25 years and above) (Fig 4b_1_–4d_1_). The pattern of fertility observed among all men in each age group was similar to that of monogamist men (Fig 4b_2_–4d_2_) except for monogamist men in the age group 15–24 years (Fig 4b_2_).

**Fig 5:**
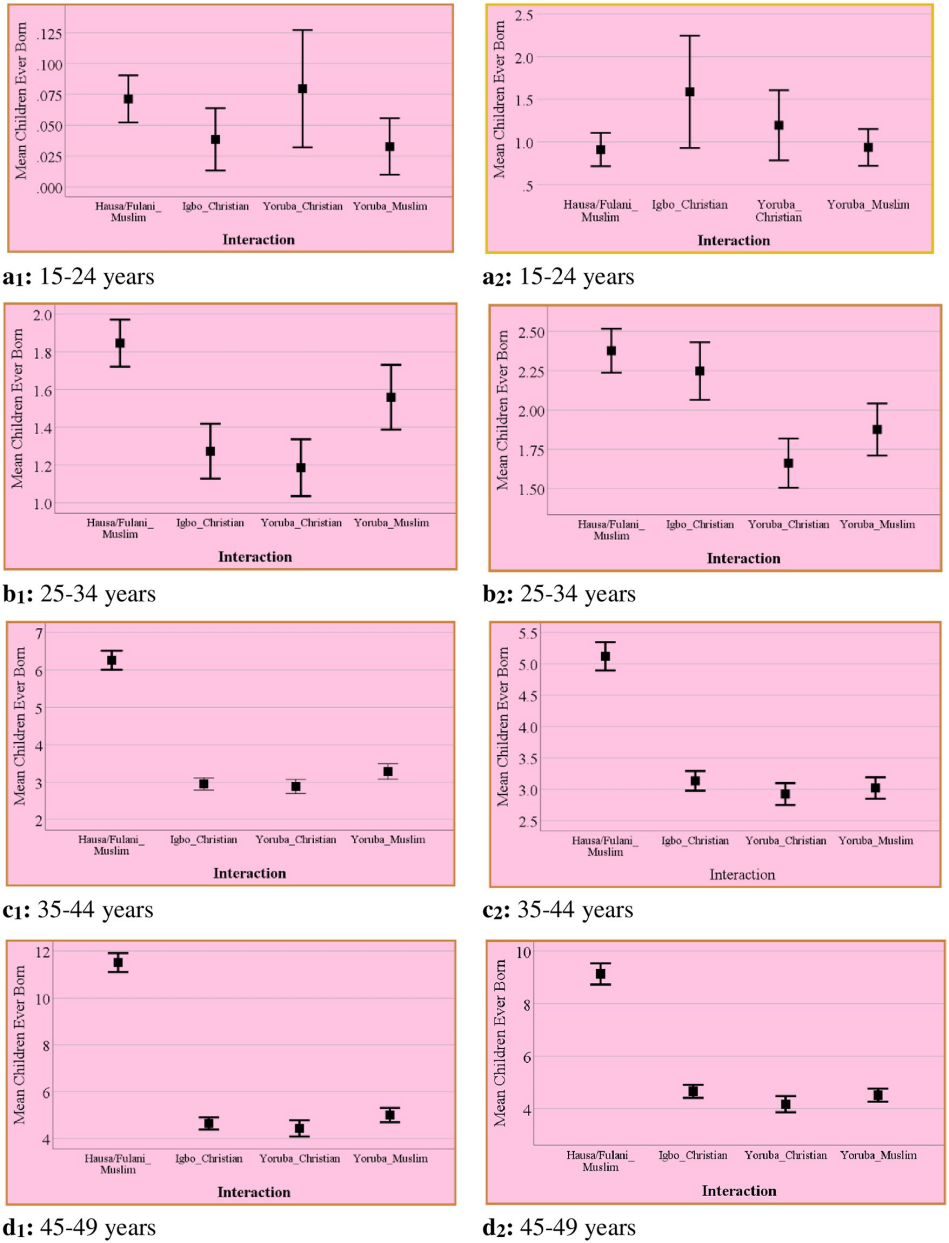
Mean Children Ever Born by Age in Years according to Religion and Ethnic Interaction (All Men (5a_1_-5d_1_) and Monogamist Men (5a_2_-5d_2_)).

The distribution of all men by mean children ever born according to REI standardized across age groups is presented in Fig 5a1–5d1. Using mean as the point estimate of CEB, the data revealed that Hausa/Fulani Muslim men experienced higher CEB at ages 25 years of age than other REI categories. In the age group 45–59 years, the mCEB was highest among Hausa/Fulani Muslim (11.51±5.97, 95% C.I = 11.10–11.91) compared to Igbo Christian (4.65±2.82, 95% C.I = 4.39–4.91), Yoruba Christian (4.44±2.67, 95% C.I = 4.09–4.78), and Yoruba Muslim (5.01±2.54, 95% C.I = 4.70–5.31). The overall mCEB for all men in Nigeria was 7.88±5.67, 95% C.I = 6.23–10.05. The pattern of mCEB observed among all men in each age group was similar (slight discrepancies in age groups 15–24 years and 25–34 years) to that of monogamist men as presented in Fig 5b_2_–5d_2_.

Table 2 presents the negative binomial model of the relationship between REI and male fertility. Four models (Models 1A-3A, and Full Model_A_) were used to describe this relationship. In Model 1A: The Incidence Rate Ratio (IRR) of fertility was higher among men who are Hausa/Fulani Muslims (IRR = 1.49, 95% C.I = 1.38–1.61, p<0.001), but less in men who are Igbo Christian (IRR = 0.88, 95% C.I = 0.81–0.96, p<0.01), Yoruba Christian (IRR = 0.84, 95% C.I = 0.76–0.93, p<0.01) than Yoruba Muslim. The IRR was found to be higher in rural areas, in polygamous families, and among those who have been married more than once. The IRR reduces with an increase in age, and an increase in the level of education, but reduces among the richer men. The pattern observed by all the variables included in Model 1A was similar to that which was found in Model 2A, Model 3A, and Full Model where other variables were included in the model. In the full model, the fertility IRR was higher among men who are Hausa/Fulani Muslims (IRR = 1.34, 95% C.I = 1.18–1.51, p<0.001), Igbo Christian (IRR = 1.18, 95% C.I = 1.05–1.31, p<0.01), but was not significantly different among Yoruba Christian (IRR = 0.98, 95% C.I = 0.87–1.12, p>0.05) than Yoruba Muslim.

**Table 2:** Negative binomial model of the relationship between ethnic religion interaction and male fertility (All MEN aged 15–59 years).

Background Characteristics	Model 1A	Model 2A	Model 3A	Full Model_A_
	uIRR(95% C.I)	aIRR(95% C.I)	aIRR(95% C.I)	aIRR(95% C.I)
**Ethnic Religion Interaction**				
Hausa/Ful. Muslim	1.49(1.38–1.61)*	1.38(1.26–1.52)*	1.34(1.20–1.48)*	1.34 (1.18–1.51)*
Igbo Christian	0.88(0.81–0.96)**	0.87(0.78–0.96)**	1.16(1.03–1.29)**	1.18(1.05–1.31)**
Yoruba Christian	0.84(0.76–0.93)**	0.92(0.82–1.03)	0.92(0.81–1.04)	0.98(0.87–1.12)
Yoruba Muslim	*Ref*.	*Ref*.	*Ref*.	*Ref*.
**Age**				
15–24	0.009(0.008–0.02)*	0.008(0.007–0.01)*		0.16(0.12–0.21)*
25–34	0.21(0.19–0.22)*	0.21(0.19–0.23)*		0.33(0.29–0.36)*
35–44	0.57(0.54–0.61)*	0.61(0.56–0.65)*		0.62(0.57–0.68)*
45–59	*Ref*.	*Ref*.		*Ref*.
**Place of Residence**				
Urban	0.71(0.68–0.74)*	0.87(0.80–0.93)*		0.99(0.91–1.08)
Rural	*Ref*.	*Ref*.		*Ref*.
**Level of Education**				
None	1.97(1.85–2.09)*	1.43(1.29–1.59)*		1.11(0.96–1.27)
Primary	1.75(1.63–1.87)*	1.42(1.29–1.58)*		1.17(1.03–1.33)***
Secondary	0.83(0.78–0.88)*	1.24(1.14–1.35)*		1.10(1.00–1.22)
Higher	*Ref*.	*Ref*.		*Ref*.
**Wealth Index**				
Poor	1.74(1.66–1.82)*	1.24(1.13–1.37)*		1.13(0.99–1.27)
Middle	1.45(1.38–1.52)*	1.26(1.16–1.36)*		1.12(1.02–1.23)***
Rich	*Ref*.	*Ref*.		*Ref*.
**Age at First Birth**				
<25	1.88(1.73–2.04)*		1.32(1.14–1.52)*	1.69(1.46–1.97)*
25–34	1.39(1.29–1.51)*		1.22(1.08–1.38)**	1.43(1.26–1.62)*
35–59	*Ref*.		*Ref*.	*Ref*.
**Marital Status**				
Never Mar.	0.008(0.006–0.01)*			
Currently Mar.	1.64(1.37–1.96)*			
Previously Mar.	*Ref*.			
**Age at First Cohabitation**				
<25	1.45(1.33–1.57)*		1.02(0.88–1.19)	1.36(1.16–1.59)*
25–34	1.13(1.04–1.23)**		0.97(0.84–1.11)	1.11(0.97–1.28)
35–59	*Ref*.		*Ref*.	*Ref*.
**Number of Marital Unions**				
Once	0.51(0.46–0.55)*		0.58(0.52–0.64)*	0.76(0.68–0.85)*
More than once	*Ref*.		*Ref*.	*Ref*.
**Family Type**				
Monogamy	0.38(0.35–0.40)*			
Polygamy	*Ref*.			
**Log Likelihood**		-15981.3	-11114.6	-10811.4
**AIC**		31988.6	22247.2	21658.8
**BIC**		32081.0	22304.7	21773.9

Ref. Reference Category; AIC: Akaike’s Information Criterion; BIC: Bayesian Information Criterion;

*Significant at 0.1%;

**Significant at 1.0%;

***Significant at 5.0%; IRR: Incidence Rate Ratio

Other factors influencing male fertility are age, level of education, wealth index, age at first birth, age at first cohabitation, and the number of marital unions. The IRR of fertility was 1.17(95% C.I = 1.03–1.33, p<0.05) and 1.12(95% C.I = 1.02–1.23, p<0.05) times higher among men who have attained primary level of education compared to their counterparts with a higher level of education and those who are in the middle wealth quintile class than the rich men respectfully. The fertility IRR was 0.16(95% C.I = 0.12–0.21, p<0.001), 0.33(95% C.I = 0.29–0.36, p<0.001), 0.62(95% C.I = 0.57–0.68, p<0.001) times higher among men aged 15–24 years, 25–34 years, 35–44 years than men in the age group 45–59 years.

The negative binomial model of the relationship between ethnic religion interaction and male fertility among only monogamous men aged 15–59 years is presented in Table 3. The data show that there were no significant differences in the fertility IRR of Yoruba Christians and Yoruba Muslims, but the fertility IRR was significantly higher among the Hausa/Fulani Muslims (IRR = 3.22. 95% C.I = 2.95–3.51. p<0.001) and Igbo Christian (IRR = 1.51. 95% C.I = 1.36–1.66. p<0.001) compared to Yoruba Muslim (Model 1B). This pattern was consistently sustained when other factors were included in the model at different levels of analysis (Model 1B, Model 2B, Model 3B, and Full Model B).

**Table 3:** Negative binomial model of the relationship between ethnic religion interaction and male fertility (Monogamist MEN aged 15–59 years).

Background Characteristics	Model 1B	Model 2B	Model 3B	Full Model_B_
	uIRR(95% C.I)	aIRR(95% C.I)	aIRR(95% C.I)	aIRR(95% C.I)
**Ethnic Religion Interaction**				
Hausa/Ful. Muslim	3.22(2.95–3.51)*	1.42(1.26–1.59)*	1.34(1.20–1.48)*	1.34(1.18–1.51)*
Igbo Christian	1.51(1.36–1.66)*	1.04(0.93–1.16)	1.16(1.03–1.29)**	1.18(1.05–1.31)**
Yoruba Christian	1.10(0.98–1.22)	0.97(0.86–1.10)	0.92(0.81–1.04)	0.99(0.86–1.12)
Yoruba Muslim	*Ref*.	*Ref*.	*Ref*.	*Ref*.
**Age**				
15–24	0.16(0.13–0.21)*	0.13(0.10–0.17)*		0.16(0.12–0.21)*
25–34	0.36(0.32–0.39)*	0.34(0.31–0.38)*		0.33(0.29–0.36)*
35–44	0.62(0.57–0.67)*	0.64(0.59–0.70)*		0.62(0.57–0.68)*
45–59	*Ref*.	*Ref*.		*Ref*.
**Place of Residence**				
Urban	0.77(0.722–0.821)*	0.97(0.89–1.05)		0.99(0.91–1.08)
Rural	*Ref*.	*Ref*.		*Ref*.
**Level of Education**				
None	1.70(1.54–1.88)*	1.24(1.08–1.41)**		1.11(0.96–1.27)
Primary	1.51(1.35–1.68)*	1.29(1.14–1.45)*		1.17(1.03–1.33)***
Secondary	1.14(1.03–1.25)**	1.22(1.11–1.35)*		1.11(1.00–1.22)
Higher	*Ref*.	*Ref*.		*Ref*.
**Wealth Index**				
Poor	1.61(1.49–1.74)*	1.24(1.09–1.40)*		1.13(0.99–1.27)
Middle	1.34(1.23–1.45)*	1.21(1.10–1.32)*		1.12(1.02–1.23)***
Rich	*Ref*.	*Ref*.		*Ref*.
**Age at First Birth**				
<25	1.62(1.45–1.80)*		1.32(1.14–1.52)*	1.70(1.46–1.97)*
25–34	1.29(1.17–1.42)*		1.22(1.08–1.38)**	1.43(1.26–1.62)*
35–59	*Ref*.		*Ref*.	*Ref*.
**Age at First Cohabitation**				
<25	1.45(1.29–1.62)*		1.02(0.88–1.19)	1.36(1.16–1.59)*
25–34	1.16(1.04–1.29)**		0.97(0.84–1.11)	1.11(0.96–1.28)
35–59	*Ref*.		*Ref*.	*Ref*.
**Number of Marital Unions**				
Once	0.486(0.44–0.53)*		0.58(0.52–0.64)*	0.76(0.68–0.85)*
More than once	*Ref*.		*Ref*.	*Ref*.
**Log Likelihood**		-11270.839	-11114.585	-10811.432
**AIC**		22567.677	22247.170	21658.863
**BIC**		22651.627	22304.728	21773.978

Ref. Reference Category; AIC: Akaike’s Information Criterion; BIC: Bayesian Information Criterion;

*Significant at 0.1%;

**Significant at 1.0%;

***Significant at 5.0%; IRR: Incidence Rate Ratio

## Discussion

A rapid increase in the population growth in Nigeria amidst poverty and dwindling economy pose grave challenges to the well-being of individuals in the country. Nigeria, the most populous country in Africa is divided by religious and ethnic lines, but not administratively structured according to this stratification. However, most sociocultural, and political situations interface with these identities to shape its population growth rate trajectory [19]. Therefore, it is undisputable to say that religion and ethnicity are influential to fertility, a primary agent of population dynamics in any Nation. We found high fertility (CEB≥5) to be more prominent among the Hausa/Fulani Muslim men than their counterparts who are Igbo Christian or Yoruba Christian or Yoruba Muslim. While the Igbo Christians and Yoruba Christians exhibit similar pattern of high fertility experience, the level was higher among Yoruba Muslims. Polygamy marriage which is more common among Nigerian Muslims than Christians can explain the high fertility difference between the Hausa Fulani Muslim and other REI groups [21]. The striking variation in high fertility between the Hausa/Fulani Muslim and Yoruba Muslims, despite being in the same religious denomination may be attributed to higher literacy level and contraceptive prevalence rate among the Yorubas than Hausa/Fulani men [22–24].

Age has a strong positive association with fertility [10, 14, 15]. Naturally, the length of exposure to the risk of childbearing increases with age. Accordingly, the incidence of male fertility increases with age [14, 25], all things being equal. Notwithstanding, socioeconomic differences, marriage postponement, material needs, and vision to attain desired career goals can intervene in the relationship between age and male fertility. Thus, the need to standardize age becomes important to the analysis of male fertility. In this study, the age-standardized mCEB by REI shows that Hausa/Fulani Muslim men have a higher proportion of their members having more children than any other REI groups and the variation was more pronounced in men aged 25 years and above. Restricting the analysis to men aged 45–59 years, the mCEB of above eleven observed among Hausa/Fulani Muslims was the highest, while slightly about four was found among Igbo Christian and Yoruba Christian, the mCEB for Yoruba Muslims was approximately five, while at the national level, the mCEB was about eight. Thus, the national estimate of mCEB was inflated by the Hausa/Fulani Muslim childbearing practices. These findings are consistent with the outcome of studies conducted among Nigerian women [11, 12]. Strong adherence to the tenets of religious and ethnic groups in Nigeria may be a possible reason for our findings. The Yoruba and Igbo Christians have a preference for monogamy type of family, while polygamy is the situation for the majority of men from the Hausa/Fulani Muslim religious background. Besides, a striking majority of Yoruba and Igbo Christians live in the Southern part of Nigeria where fertility control and childbearing delay mechanisms are most prevalent in the country [21, 22].

The unadjusted NB model showed that compared to Yoruba Muslims, being Hausa/Fulani Muslim predisposed men to greater fertility IRR, while lower IRR was experienced by the Igbo Christians and Yoruba Christians. However, at the multivariate level of analysis, the pattern exhibited by the Hausa/Fulani Muslims compared to Yoruba Muslims was sustained but, a reverse pattern was observed among Igbo Christians compared to Yoruba Muslims. At this level of analysis, no significant difference in male fertility was found among the Yoruba Muslims and Yoruba Christians. Higher IRR among the Hausa/Fulani Muslims and Yoruba Muslims can be ascribed to the differences in the level of education, wealth, social class, lifestyle, and other characteristics [17]. Aside from REI, the other key determinants of male fertility found in this study are age, level of education, wealth index, age at first birth, age at first cohabitation, and the number of marital unions. These male fertility predictors have been established by earlier studies [14, 25–27] and the pattern is similar to well-documented female fertility research in Nigeria and elsewhere [9, 10, 28].

Restricting the analyzed data to only monogamist men revealed no significant differences in the fertility IRR of Yoruba Christian and Yoruba Muslim, but the fertility IRR was significantly higher among the Hausa/Fulani Muslim and Igbo Christian than the Yoruba Muslim men. Higher fertility has been consistently reported in the core-north part of Nigeria which is predominantly populated by Hausa/Fulani Muslims compared to the southwest geopolitical zone dominated by the Yorubas. The observed higher fertility experience among Hausa/Fulani Muslim men than Yoruba Muslim men in monogamous families could be attributed to the higher literacy level in the south-west which has eroded some cultural identities that promote fertility including early marriage, early childbearing, family size, etc. irrespective of their religious background [5, 11]. Time spent on schooling and contraceptive adoption are other possible explanations for this difference. The higher fertility found among Igbo Christians than Yoruba Muslim men in monogamous families cannot be isolated from a high preference for a male child and low uptake of modern contraceptives among the Igbo ethnic group [10–12].

More cultural factors that can modify the relationship between REI and male fertility in Nigeria were not included in the original questionnaire used to collect the data for this study. The cross-sectional nature of this data where the current religion practiced by men was used in the data analysis may have a slight influence on the output of this study. This is because the religious practice of individuals might have changed in the course of the family-building process. Although, only in the rare situation that Nigerian men experience a religious switch, the readers of this article are alerted to interpret the findings of this study with caution. The robust and nationally representative sample employed in this study remains its strength. Besides, while uniform policies often emerge from fertility programs that aim at meeting national requirements, the success of such policies depends upon the degree of variability in the relevant behaviors across religious groups within a particular ethnic line. Therefore, assessing the variation in fertility across religious groups along the ethnic line is a major contribution to knowledge on the association between ethnicity, religion, and male fertility in Nigeria.

## Conclusions

We conclude that REI plays an influential role in male fertility in Nigeria. The level of male fertility is high in Nigeria, but disparity exists across the REI groups with the Hausa/Fulani Muslims being the major contributors to the observed level of male fertility. The outcome of this study is consistent with the cultural hypothesis, because the statistical relationship between male fertility and REI persists, even after controlling for selected socio-demographic characteristics. REI was an important determinant of male fertility in Nigeria, age, level of education, wealth index, age at first birth, age at first cohabitation, and the number of marital unions is other factors. While the population control agencies are encouraged to consider these identified determinants of male fertility in their fertility reduction strategy in Nigeria, more attention should be devoted to bridging the gap in access to fertility control measures and programmes that might have resulted from religion and ethnic differences in male fertility in Nigeria.
